# Supplementary material for: Quantifying spatio-temporal variation in aquaculture production areas in Satkhira, Bangladesh using geospatial and social survey
Source: PLoS One. 2022 Dec 15;17(12):e0278042. doi: 10.1371/journal.pone.0278042 (PMC9754591; doi:10.1371/journal.pone.0278042)
Supplement: S1 Table — (PDF) [file pone.0278042.s001.pdf]

Supplementary table S1

List of Sentinel-2 data acquisition

**Date of Acquisition of Sentinel-2 data**	**Sentinel-2 data Scene ID**
2017/1/2	COPERNICUS/S2/20170102T043152_20170102T043712_T45QXE
	COPERNICUS/S2/20170102T043152_20170102T043712_T45QYE
	COPERNICUS/S2/20170102T043152_20170102T043712_T45QYF
2017/1/15	COPERNICUS/S2/20170115T044121_20170115T044124_T45QXE
	COPERNICUS/S2/20170115T044121_20170115T044124_T45QXF
	COPERNICUS/S2/20170115T044121_20170115T044124_T45QYE
	COPERNICUS/S2/20170115T044121_20170115T044124_T45QYF
2017/1/25	COPERNICUS/S2/20170125T044051_20170125T044345_T45QXE
	COPERNICUS/S2/20170125T044051_20170125T044345_T45QXF
	COPERNICUS/S2/20170125T044051_20170125T044345_T45QYE
	COPERNICUS/S2/20170125T044051_20170125T044345_T45QYF
2017/2/1	COPERNICUS/S2/20170201T043011_20170201T043825_T45QXE
	COPERNICUS/S2/20170201T043011_20170201T043825_T45QYE
	COPERNICUS/S2/20170201T043011_20170201T043825_T45QYF
2017/2/4	COPERNICUS/S2/20170204T044001_20170204T044509_T45QXE
	COPERNICUS/S2/20170204T044001_20170204T044509_T45QXF
	COPERNICUS/S2/20170204T044001_20170204T044509_T45QYE
	COPERNICUS/S2/20170204T044001_20170204T044509_T45QYF
2017/2/11	COPERNICUS/S2/20170211T042911_20170211T043113_T45QXE
	COPERNICUS/S2/20170211T042911_20170211T043113_T45QYE
	COPERNICUS/S2/20170211T042911_20170211T043113_T45QYF
2017/2/24	COPERNICUS/S2/20170224T043751_20170224T044228_T45QXE
	COPERNICUS/S2/20170224T043751_20170224T044228_T45QXF
	COPERNICUS/S2/20170224T043751_20170224T044228_T45QYE
	COPERNICUS/S2/20170224T043751_20170224T044228_T45QYF
2017/3/3	COPERNICUS/S2/20170303T042701_20170303T043315_T45QXE
	COPERNICUS/S2/20170303T042701_20170303T043315_T45QYE
	COPERNICUS/S2/20170303T042701_20170303T043315_T45QYF
2017/3/16	COPERNICUS/S2/20170316T043651_20170316T044657_T45QXE
	COPERNICUS/S2/20170316T043651_20170316T044657_T45QXF
	COPERNICUS/S2/20170316T043651_20170316T044657_T45QYE
	COPERNICUS/S2/20170316T043651_20170316T044657_T45QYF
2017/3/23	COPERNICUS/S2/20170323T042701_20170323T043930_T45QXE
	COPERNICUS/S2/20170323T042701_20170323T043930_T45QYE
	COPERNICUS/S2/20170323T042701_20170323T043930_T45QYF
2017/12/1	COPERNICUS/S2/20171201T044131_20171201T044605_T45QXE
	COPERNICUS/S2/20171201T044131_20171201T044605_T45QXF

	COPERNICUS/S2/20171201T044131_20171201T044605_T45QYE
	COPERNICUS/S2/20171201T044131_20171201T044605_T45QYF
2017/12/8	COPERNICUS/S2/20171208T043141_20171208T043944_T45QXE
	COPERNICUS/S2/20171208T043141_20171208T043944_T45QYE
	COPERNICUS/S2/20171208T043141_20171208T043944_T45QYF
2017/12/13	COPERNICUS/S2/20171213T043139_20171213T043942_T45QXE
	COPERNICUS/S2/20171213T043139_20171213T043942_T45QYE
	COPERNICUS/S2/20171213T043139_20171213T043942_T45QYF
2017/12/16	COPERNICUS/S2/20171216T044159_20171216T045011_T45QXE
	COPERNICUS/S2/20171216T044159_20171216T045011_T45QXF
	COPERNICUS/S2/20171216T044159_20171216T045011_T45QYE
	COPERNICUS/S2/20171216T044159_20171216T045011_T45QYF
2017/12/21	COPERNICUS/S2/20171221T044211_20171221T044710_T45QXE
	COPERNICUS/S2/20171221T044211_20171221T044710_T45QXF
	COPERNICUS/S2/20171221T044211_20171221T044710_T45QYE
	COPERNICUS/S2/20171221T044211_20171221T044710_T45QYF
2017/12/23	COPERNICUS/S2/20171223T043159_20171223T043613_T45QXE
	COPERNICUS/S2/20171223T043159_20171223T043613_T45QYE
	COPERNICUS/S2/20171223T043159_20171223T043613_T45QYF
2017/12/26	COPERNICUS/S2/20171226T044159_20171226T044201_T45QXE
	COPERNICUS/S2/20171226T044159_20171226T044201_T45QXF
	COPERNICUS/S2/20171226T044159_20171226T044201_T45QYE
	COPERNICUS/S2/20171226T044159_20171226T044201_T45QYF
2017/12/28	COPERNICUS/S2/20171228T043201_20171228T044000_T45QXE
	COPERNICUS/S2/20171228T043201_20171228T044000_T45QYE
	COPERNICUS/S2/20171228T043201_20171228T044000_T45QYF
2018/1/2	COPERNICUS/S2/20180102T043149_20180102T043943_T45QXE
	COPERNICUS/S2/20180102T043149_20180102T043943_T45QYE
	COPERNICUS/S2/20180102T043149_20180102T043943_T45QYF
2018/1/5	COPERNICUS/S2/20180105T044149_20180105T045004_T45QXE
	COPERNICUS/S2/20180105T044149_20180105T045004_T45QXF
	COPERNICUS/S2/20180105T044149_20180105T045004_T45QYE
	COPERNICUS/S2/20180105T044149_20180105T045004_T45QYF
2018/1/10	COPERNICUS/S2/20180110T044141_20180110T044352_T45QXE
	COPERNICUS/S2/20180110T044141_20180110T044352_T45QXF
	COPERNICUS/S2/20180110T044141_20180110T044352_T45QYE
	COPERNICUS/S2/20180110T044141_20180110T044352_T45QYF
2018/1/12	COPERNICUS/S2/20180112T043129_20180112T043557_T45QXE
	COPERNICUS/S2/20180112T043129_20180112T043557_T45QYE
	COPERNICUS/S2/20180112T043129_20180112T043557_T45QYF
2018/1/15	COPERNICUS/S2/20180115T044129_20180115T044928_T45QXE

	COPERNICUS/S2/20180115T044129_20180115T044928_T45QXF
	COPERNICUS/S2/20180115T044129_20180115T044928_T45QYE
	COPERNICUS/S2/20180115T044129_20180115T044928_T45QYF
2018/1/17	COPERNICUS/S2/20180117T043121_20180117T043916_T45QXE
	COPERNICUS/S2/20180117T043121_20180117T043916_T45QYE
	COPERNICUS/S2/20180117T043121_20180117T043916_T45QYF
2018/1/22	COPERNICUS/S2/20180122T043059_20180122T043855_T45QXE
	COPERNICUS/S2/20180122T043059_20180122T043855_T45QYE
	COPERNICUS/S2/20180122T043059_20180122T043855_T45QYF
2018/1/25	COPERNICUS/S2/20180125T044049_20180125T044829_T45QXE
	COPERNICUS/S2/20180125T044049_20180125T044829_T45QXF
	COPERNICUS/S2/20180125T044049_20180125T044829_T45QYE
	COPERNICUS/S2/20180125T044049_20180125T044829_T45QYF
2018/1/30	COPERNICUS/S2/20180130T044031_20180130T045100_T45QXE
	COPERNICUS/S2/20180130T044031_20180130T045100_T45QXF
	COPERNICUS/S2/20180130T044031_20180130T045100_T45QYE
	COPERNICUS/S2/20180130T044031_20180130T045100_T45QYF
2018/2/1	COPERNICUS/S2/20180201T043009_20180201T043008_T45QXE
	COPERNICUS/S2/20180201T043009_20180201T043008_T45QYE
	COPERNICUS/S2/20180201T043009_20180201T043008_T45QYF
2018/2/4	COPERNICUS/S2/20180204T043959_20180204T044709_T45QXE
	COPERNICUS/S2/20180204T043959_20180204T044709_T45QXF
	COPERNICUS/S2/20180204T043959_20180204T044709_T45QYE
	COPERNICUS/S2/20180204T043959_20180204T044709_T45QYF
2018/2/6	COPERNICUS/S2/20180206T042941_20180206T043609_T45QXE
	COPERNICUS/S2/20180206T042941_20180206T043609_T45QYE
	COPERNICUS/S2/20180206T042941_20180206T043609_T45QYF
2018/2/9	COPERNICUS/S2/20180209T043931_20180209T043929_T45QXE
	COPERNICUS/S2/20180209T043931_20180209T043929_T45QXF
	COPERNICUS/S2/20180209T043931_20180209T043929_T45QYE
	COPERNICUS/S2/20180209T043931_20180209T043929_T45QYF
2018/2/11	COPERNICUS/S2/20180211T042909_20180211T043709_T45QXE
	COPERNICUS/S2/20180211T042909_20180211T043709_T45QYE
	COPERNICUS/S2/20180211T042909_20180211T043709_T45QYF
2018/2/14	COPERNICUS/S2/20180214T043859_20180214T044445_T45QXE
	COPERNICUS/S2/20180214T043859_20180214T044445_T45QXF
	COPERNICUS/S2/20180214T043859_20180214T044445_T45QYE
	COPERNICUS/S2/20180214T043859_20180214T044445_T45QYF
2018/2/16	COPERNICUS/S2/20180216T042841_20180216T042842_T45QXE
	COPERNICUS/S2/20180216T042841_20180216T042842_T45QYE
	COPERNICUS/S2/20180216T042841_20180216T042842_T45QYF

2018/2/19	COPERNICUS/S2/20180219T043831_20180219T044333_T45QXE
	COPERNICUS/S2/20180219T043831_20180219T044333_T45QXF
	COPERNICUS/S2/20180219T043831_20180219T044333_T45QYE
	COPERNICUS/S2/20180219T043831_20180219T044333_T45QYF
2018/2/21	COPERNICUS/S2/20180221T042809_20180221T042803_T45QXE
	COPERNICUS/S2/20180221T042809_20180221T042803_T45QYE
	COPERNICUS/S2/20180221T042809_20180221T042803_T45QYF
2018/2/24	COPERNICUS/S2/20180224T043749_20180224T045147_T45QXE
	COPERNICUS/S2/20180224T043749_20180224T045147_T45QXF
	COPERNICUS/S2/20180224T043749_20180224T045147_T45QYE
	COPERNICUS/S2/20180224T043749_20180224T045147_T45QYF
2018/2/26	COPERNICUS/S2/20180226T042731_20180226T043323_T45QXE
	COPERNICUS/S2/20180226T042731_20180226T043323_T45QYE
	COPERNICUS/S2/20180226T042731_20180226T043323_T45QYF
2018/3/1	COPERNICUS/S2/20180301T043721_20180301T044244_T45QXE
	COPERNICUS/S2/20180301T043721_20180301T044244_T45QXF
	COPERNICUS/S2/20180301T043721_20180301T044244_T45QYE
	COPERNICUS/S2/20180301T043721_20180301T044244_T45QYF
2018/3/3	COPERNICUS/S2/20180303T042659_20180303T043038_T45QXE
	COPERNICUS/S2/20180303T042659_20180303T043038_T45QYE
	COPERNICUS/S2/20180303T042659_20180303T043038_T45QYF
2018/3/6	COPERNICUS/S2/20180306T043649_20180306T044806_T45QXE
	COPERNICUS/S2/20180306T043649_20180306T044806_T45QXF
	COPERNICUS/S2/20180306T043649_20180306T044806_T45QYE
	COPERNICUS/S2/20180306T043649_20180306T044806_T45QYF
2018/3/8	COPERNICUS/S2/20180308T042701_20180308T043336_T45QXE
	COPERNICUS/S2/20180308T042701_20180308T043336_T45QYE
	COPERNICUS/S2/20180308T042701_20180308T043336_T45QYF
2018/3/11	COPERNICUS/S2/20180311T043701_20180311T044818_T45QXE
	COPERNICUS/S2/20180311T043701_20180311T044818_T45QXF
	COPERNICUS/S2/20180311T043701_20180311T044818_T45QYE
	COPERNICUS/S2/20180311T043701_20180311T044818_T45QYF
2018/3/13	COPERNICUS/S2/20180313T042659_20180313T043611_T45QXE
	COPERNICUS/S2/20180313T042659_20180313T043611_T45QYE
	COPERNICUS/S2/20180313T042659_20180313T043611_T45QYF
2018/3/16	COPERNICUS/S2/20180316T043659_20180316T044102_T45QXE
	COPERNICUS/S2/20180316T043659_20180316T044102_T45QXF
	COPERNICUS/S2/20180316T043659_20180316T044102_T45QYE
	COPERNICUS/S2/20180316T043659_20180316T044102_T45QYF
2018/3/18	COPERNICUS/S2/20180318T042701_20180318T044053_T45QXE
	COPERNICUS/S2/20180318T042701_20180318T044053_T45QYE

	COPERNICUS/S2/20180318T042701_20180318T044053_T45QYF
2018/3/21	COPERNICUS/S2/20180321T043701_20180321T044259_T45QXE
	COPERNICUS/S2/20180321T043701_20180321T044259_T45QXF
	COPERNICUS/S2/20180321T043701_20180321T044259_T45QYE
	COPERNICUS/S2/20180321T043701_20180321T044259_T45QYF
2018/3/23	COPERNICUS/S2/20180323T042659_20180323T044040_T45QXE
	COPERNICUS/S2/20180323T042659_20180323T044040_T45QYE
	COPERNICUS/S2/20180323T042659_20180323T044040_T45QYF
2018/3/26	COPERNICUS/S2/20180326T043659_20180326T043753_T45QXE
	COPERNICUS/S2/20180326T043659_20180326T043753_T45QXF
	COPERNICUS/S2/20180326T043659_20180326T043753_T45QYE
	COPERNICUS/S2/20180326T043659_20180326T043753_T45QYF
2018/3/28	COPERNICUS/S2/20180328T042701_20180328T044110_T45QXE
	COPERNICUS/S2/20180328T042701_20180328T044110_T45QYE
	COPERNICUS/S2/20180328T042701_20180328T044110_T45QYF
2018/12/1	COPERNICUS/S2/20181201T044139_20181201T044801_T45QXE
	COPERNICUS/S2/20181201T044139_20181201T044801_T45QXF
	COPERNICUS/S2/20181201T044139_20181201T044801_T45QYE
	COPERNICUS/S2/20181201T044139_20181201T044801_T45QYF
2018/12/3	COPERNICUS/S2/20181203T043131_20181203T043911_T45QXE
	COPERNICUS/S2/20181203T043131_20181203T043911_T45QYE
	COPERNICUS/S2/20181203T043131_20181203T043911_T45QYF
2018/12/6	COPERNICUS/S2/20181206T044141_20181206T044630_T45QXE
	COPERNICUS/S2/20181206T044141_20181206T044630_T45QXF
	COPERNICUS/S2/20181206T044141_20181206T044630_T45QYE
	COPERNICUS/S2/20181206T044141_20181206T044630_T45QYF
2018/12/11	COPERNICUS/S2/20181211T044159_20181211T044627_T45QXE
	COPERNICUS/S2/20181211T044159_20181211T044627_T45QXF
	COPERNICUS/S2/20181211T044159_20181211T044627_T45QYE
	COPERNICUS/S2/20181211T044159_20181211T044627_T45QYF
2018/12/13	COPERNICUS/S2/20181213T043151_20181213T043713_T45QXE
	COPERNICUS/S2/20181213T043151_20181213T043713_T45QYE
	COPERNICUS/S2/20181213T043151_20181213T043713_T45QYF
2018/12/16	COPERNICUS/S2/20181216T044201_20181216T044230_T45QXE
	COPERNICUS/S2/20181216T044201_20181216T044230_T45QXF
	COPERNICUS/S2/20181216T044201_20181216T044230_T45QYE
	COPERNICUS/S2/20181216T044201_20181216T044230_T45QYF
2018/12/18	COPERNICUS/S2/20181218T043159_20181218T043722_T45QXE
	COPERNICUS/S2/20181218T043159_20181218T043722_T45QYE
	COPERNICUS/S2/20181218T043159_20181218T043722_T45QYF
2018/12/21	COPERNICUS/S2/20181221T044209_20181221T044653_T45QXE

	COPERNICUS/S2/20181221T044209_20181221T044653_T45QXF
	COPERNICUS/S2/20181221T044209_20181221T044653_T45QYE
	COPERNICUS/S2/20181221T044209_20181221T044653_T45QYF
2018/12/23	COPERNICUS/S2/20181223T043201_20181223T043736_T45QXE
	COPERNICUS/S2/20181223T043201_20181223T043736_T45QYE
	COPERNICUS/S2/20181223T043201_20181223T043736_T45QYF
2018/12/26	COPERNICUS/S2/20181226T044211_20181226T044206_T45QXE
	COPERNICUS/S2/20181226T044211_20181226T044206_T45QXF
	COPERNICUS/S2/20181226T044211_20181226T044206_T45QYE
	COPERNICUS/S2/20181226T044211_20181226T044206_T45QYF
2018/12/28	COPERNICUS/S2/20181228T043209_20181228T043927_T45QXE
	COPERNICUS/S2/20181228T043209_20181228T043927_T45QYE
	COPERNICUS/S2/20181228T043209_20181228T043927_T45QYF
2019/1/2	COPERNICUS/S2/20190102T043151_20190102T043514_T45QXE
	COPERNICUS/S2/20190102T043151_20190102T043514_T45QYE
	COPERNICUS/S2/20190102T043151_20190102T043514_T45QYF
2019/1/5	COPERNICUS/S2/20190105T044201_20190105T044654_T45QXE
	COPERNICUS/S2/20190105T044201_20190105T044654_T45QXF
	COPERNICUS/S2/20190105T044201_20190105T044654_T45QYE
	COPERNICUS/S2/20190105T044201_20190105T044654_T45QYF
2019/1/7	COPERNICUS/S2/20190107T043149_20190107T043708_T45QXE
	COPERNICUS/S2/20190107T043149_20190107T043708_T45QYE
	COPERNICUS/S2/20190107T043149_20190107T043708_T45QYF
2019/1/10	COPERNICUS/S2/20190110T044149_20190110T044556_T45QXE
	COPERNICUS/S2/20190110T044149_20190110T044556_T45QXF
	COPERNICUS/S2/20190110T044149_20190110T044556_T45QYE
	COPERNICUS/S2/20190110T044149_20190110T044556_T45QYF
2019/1/12	COPERNICUS/S2/20190112T043141_20190112T043642_T45QXE
	COPERNICUS/S2/20190112T043141_20190112T043642_T45QYE
	COPERNICUS/S2/20190112T043141_20190112T043642_T45QYF
2019/1/15	COPERNICUS/S2/20190115T044131_20190115T044130_T45QXE
	COPERNICUS/S2/20190115T044131_20190115T044130_T45QXF
	COPERNICUS/S2/20190115T044131_20190115T044130_T45QYE
	COPERNICUS/S2/20190115T044131_20190115T044130_T45QYF
2019/1/17	COPERNICUS/S2/20190117T043119_20190117T043639_T45QXE
	COPERNICUS/S2/20190117T043119_20190117T043639_T45QYE
	COPERNICUS/S2/20190117T043119_20190117T043639_T45QYF
2019/1/20	COPERNICUS/S2/20190120T044119_20190120T044409_T45QXE
	COPERNICUS/S2/20190120T044119_20190120T044409_T45QXF
	COPERNICUS/S2/20190120T044119_20190120T044409_T45QYE
	COPERNICUS/S2/20190120T044119_20190120T044409_T45QYF

2019/1/22	COPERNICUS/S2/20190122T043101_20190122T043322_T45QXE
	COPERNICUS/S2/20190122T043101_20190122T043322_T45QYE
	COPERNICUS/S2/20190122T043101_20190122T043322_T45QYF
2019/1/25	COPERNICUS/S2/20190125T044051_20190125T044051_T45QXE
	COPERNICUS/S2/20190125T044051_20190125T044051_T45QXF
	COPERNICUS/S2/20190125T044051_20190125T044051_T45QYE
	COPERNICUS/S2/20190125T044051_20190125T044051_T45QYF
2019/1/27	COPERNICUS/S2/20190127T043039_20190127T043627_T45QXE
	COPERNICUS/S2/20190127T043039_20190127T043627_T45QYE
	COPERNICUS/S2/20190127T043039_20190127T043627_T45QYF
2019/1/30	COPERNICUS/S2/20190130T044029_20190130T044444_T45QXE
	COPERNICUS/S2/20190130T044029_20190130T044444_T45QXF
	COPERNICUS/S2/20190130T044029_20190130T044444_T45QYE
	COPERNICUS/S2/20190130T044029_20190130T044444_T45QYF
2019/2/1	COPERNICUS/S2/20190201T043011_20190201T044135_T45QXE
	COPERNICUS/S2/20190201T043011_20190201T044135_T45QYE
	COPERNICUS/S2/20190201T043011_20190201T044135_T45QYF
2019/2/4	COPERNICUS/S2/20190204T044001_20190204T045119_T45QXE
	COPERNICUS/S2/20190204T044001_20190204T045119_T45QXF
	COPERNICUS/S2/20190204T044001_20190204T045119_T45QYE
	COPERNICUS/S2/20190204T044001_20190204T045119_T45QYF
2019/2/6	COPERNICUS/S2/20190206T042949_20190206T043539_T45QXE
	COPERNICUS/S2/20190206T042949_20190206T043539_T45QYE
	COPERNICUS/S2/20190206T042949_20190206T043539_T45QYF
2019/2/9	COPERNICUS/S2/20190209T043939_20190209T045147_T45QXE
	COPERNICUS/S2/20190209T043939_20190209T045147_T45QXF
	COPERNICUS/S2/20190209T043939_20190209T045147_T45QYE
	COPERNICUS/S2/20190209T043939_20190209T045147_T45QYF
2019/2/11	COPERNICUS/S2/20190211T042921_20190211T043121_T45QXE
	COPERNICUS/S2/20190211T042921_20190211T043121_T45QYE
	COPERNICUS/S2/20190211T042921_20190211T043121_T45QYF
2019/2/14	COPERNICUS/S2/20190214T043901_20190214T045152_T45QXE
	COPERNICUS/S2/20190214T043901_20190214T045152_T45QXF
	COPERNICUS/S2/20190214T043901_20190214T045152_T45QYE
	COPERNICUS/S2/20190214T043901_20190214T045152_T45QYF
2019/2/16	COPERNICUS/S2/20190216T042849_20190216T044106_T45QXE
	COPERNICUS/S2/20190216T042849_20190216T044106_T45QYE
	COPERNICUS/S2/20190216T042849_20190216T044106_T45QYF
2019/2/19	COPERNICUS/S2/20190219T043829_20190219T044608_T45QXF
	COPERNICUS/S2/20190219T043829_20190219T044608_T45QYF
	COPERNICUS/S2/20190219T043829_20190219T045209_T45QXE

	COPERNICUS/S2/20190219T043829_20190219T045209_T45QXF
	COPERNICUS/S2/20190219T043829_20190219T045209_T45QYE
	COPERNICUS/S2/20190219T043829_20190219T045209_T45QYF
2019/2/21	COPERNICUS/S2/20190221T042811_20190221T043725_T45QXE
	COPERNICUS/S2/20190221T042811_20190221T043725_T45QYE
	COPERNICUS/S2/20190221T042811_20190221T043725_T45QYF
2019/2/24	COPERNICUS/S2/20190224T043751_20190224T044705_T45QXE
	COPERNICUS/S2/20190224T043751_20190224T044705_T45QXF
	COPERNICUS/S2/20190224T043751_20190224T044705_T45QYE
	COPERNICUS/S2/20190224T043751_20190224T044705_T45QYF
2019/2/26	COPERNICUS/S2/20190226T042739_20190226T043910_T45QXE
	COPERNICUS/S2/20190226T042739_20190226T043910_T45QYE
	COPERNICUS/S2/20190226T042739_20190226T043910_T45QYF
2019/3/1	COPERNICUS/S2/20190301T043719_20190301T044552_T45QXE
	COPERNICUS/S2/20190301T043719_20190301T044552_T45QXF
	COPERNICUS/S2/20190301T043719_20190301T044552_T45QYE
	COPERNICUS/S2/20190301T043719_20190301T044552_T45QYF
2019/3/3	COPERNICUS/S2/20190303T042701_20190303T042658_T45QXE
	COPERNICUS/S2/20190303T042701_20190303T042658_T45QYE
	COPERNICUS/S2/20190303T042701_20190303T042658_T45QYF
2019/3/6	COPERNICUS/S2/20190306T043701_20190306T044716_T45QXE
	COPERNICUS/S2/20190306T043701_20190306T044716_T45QXF
	COPERNICUS/S2/20190306T043701_20190306T044716_T45QYE
	COPERNICUS/S2/20190306T043701_20190306T044716_T45QYF
2019/3/8	COPERNICUS/S2/20190308T042659_20190308T044054_T45QXE
	COPERNICUS/S2/20190308T042659_20190308T044054_T45QYE
	COPERNICUS/S2/20190308T042659_20190308T044054_T45QYF
2019/3/11	COPERNICUS/S2/20190311T043659_20190311T044711_T45QXE
	COPERNICUS/S2/20190311T043659_20190311T044711_T45QXF
	COPERNICUS/S2/20190311T043659_20190311T044711_T45QYE
	COPERNICUS/S2/20190311T043659_20190311T044711_T45QYF
2019/3/13	COPERNICUS/S2/20190313T042701_20190313T043354_T45QXE
	COPERNICUS/S2/20190313T042701_20190313T043354_T45QYE
	COPERNICUS/S2/20190313T042701_20190313T043354_T45QYF
2019/3/16	COPERNICUS/S2/20190316T043701_20190316T044421_T45QXE
	COPERNICUS/S2/20190316T043701_20190316T044421_T45QXF
	COPERNICUS/S2/20190316T043701_20190316T044421_T45QYE
	COPERNICUS/S2/20190316T043701_20190316T044421_T45QYF
2019/3/18	COPERNICUS/S2/20190318T042659_20190318T043234_T45QXE
	COPERNICUS/S2/20190318T042659_20190318T043234_T45QYE
	COPERNICUS/S2/20190318T042659_20190318T043234_T45QYF

2019/3/21	COPERNICUS/S2/20190321T043659_20190321T044228_T45QXE
	COPERNICUS/S2/20190321T043659_20190321T044228_T45QXF
	COPERNICUS/S2/20190321T043659_20190321T044228_T45QYE
	COPERNICUS/S2/20190321T043659_20190321T044228_T45QYF
2019/3/23	COPERNICUS/S2/20190323T042701_20190323T043918_T45QXE
	COPERNICUS/S2/20190323T042701_20190323T043918_T45QYE
	COPERNICUS/S2/20190323T042701_20190323T043918_T45QYF
2019/3/26	COPERNICUS/S2/20190326T043701_20190326T045122_T45QXE
	COPERNICUS/S2/20190326T043701_20190326T045122_T45QXF
	COPERNICUS/S2/20190326T043701_20190326T045122_T45QYE
	COPERNICUS/S2/20190326T043701_20190326T045122_T45QYF
2019/3/28	COPERNICUS/S2/20190328T042709_20190328T043805_T45QXE
	COPERNICUS/S2/20190328T042709_20190328T043805_T45QYE
	COPERNICUS/S2/20190328T042709_20190328T043805_T45QYF
2019/12/1	COPERNICUS/S2/20191201T044141_20191201T044641_T45QXE
	COPERNICUS/S2/20191201T044141_20191201T044641_T45QXF
	COPERNICUS/S2/20191201T044141_20191201T044641_T45QYE
	COPERNICUS/S2/20191201T044141_20191201T044641_T45QYF
2019/12/3	COPERNICUS/S2/20191203T043139_20191203T044033_T45QXE
	COPERNICUS/S2/20191203T043139_20191203T044033_T45QYE
	COPERNICUS/S2/20191203T043139_20191203T044033_T45QYF
2019/12/6	COPERNICUS/S2/20191206T044139_20191206T045045_T45QXE
	COPERNICUS/S2/20191206T044139_20191206T045045_T45QXF
	COPERNICUS/S2/20191206T044139_20191206T045045_T45QYE
	COPERNICUS/S2/20191206T044139_20191206T045045_T45QYF
2019/12/8	COPERNICUS/S2/20191208T043151_20191208T043545_T45QXE
	COPERNICUS/S2/20191208T043151_20191208T043545_T45QYE
	COPERNICUS/S2/20191208T043151_20191208T043545_T45QYF
2019/12/11	COPERNICUS/S2/20191211T044201_20191211T044550_T45QXE
	COPERNICUS/S2/20191211T044201_20191211T044550_T45QXF
	COPERNICUS/S2/20191211T044201_20191211T044550_T45QYE
	COPERNICUS/S2/20191211T044201_20191211T044550_T45QYF
2019/12/13	COPERNICUS/S2/20191213T043149_20191213T043430_T45QXE
	COPERNICUS/S2/20191213T043149_20191213T043430_T45QYE
	COPERNICUS/S2/20191213T043149_20191213T043430_T45QYF
2019/12/16	COPERNICUS/S2/20191216T044209_20191216T044544_T45QXE
	COPERNICUS/S2/20191216T044209_20191216T044544_T45QXF
	COPERNICUS/S2/20191216T044209_20191216T044544_T45QYE
	COPERNICUS/S2/20191216T044209_20191216T044544_T45QYF
2019/12/18	COPERNICUS/S2/20191218T043201_20191218T043200_T45QXE
	COPERNICUS/S2/20191218T043201_20191218T043200_T45QYE

	COPERNICUS/S2/20191218T043201_20191218T043200_T45QYF
2019/12/21	COPERNICUS/S2/20191221T044211_20191221T044717_T45QXE
	COPERNICUS/S2/20191221T044211_20191221T044717_T45QXF
	COPERNICUS/S2/20191221T044211_20191221T044717_T45QYE
	COPERNICUS/S2/20191221T044211_20191221T044717_T45QYF
2019/12/23	COPERNICUS/S2/20191223T043209_20191223T043931_T45QXE
	COPERNICUS/S2/20191223T043209_20191223T043931_T45QYE
	COPERNICUS/S2/20191223T043209_20191223T043931_T45QYF
2019/12/26	COPERNICUS/S2/20191226T044209_20191226T044950_T45QXE
	COPERNICUS/S2/20191226T044209_20191226T044950_T45QXF
	COPERNICUS/S2/20191226T044209_20191226T044950_T45QYE
	COPERNICUS/S2/20191226T044209_20191226T044950_T45QYF
2019/12/28	COPERNICUS/S2/20191228T043201_20191228T043729_T45QXE
	COPERNICUS/S2/20191228T043201_20191228T043729_T45QYE
	COPERNICUS/S2/20191228T043201_20191228T043729_T45QYF

List of Sentinel-1 data acquisition

Date of acquisition of Sentinel-1 data	Sentinel-1 Scene ID
2017/1/2	COPERNICUS/S1_GRD/S1A_IW_GRDH_1SDV_20170102T000359_20170102T000424_014645_017D10_029D
2017/1/8	COPERNICUS/S1_GRD/S1A_IW_GRDH_1SDV_20170108T235554_20170108T235619_014747_018021_6ABB
2017/1/26	COPERNICUS/S1_GRD/S1A_IW_GRDH_1SDV_20170126T000358_20170126T000423_014995_0187CF_D86C
2017/5/2	COPERNICUS/S1_GRD/S1A_IW_GRDH_1SDV_20170502T000359_20170502T000424_016395_01B25B_8BF2
2017/5/8	COPERNICUS/S1_GRD/S1A_IW_GRDH_1SDV_20170508T235552_20170508T235617_016497_01B575_8C37
2017/5/14	COPERNICUS/S1_GRD/S1A_IW_GRDH_1SDV_20170514T000400_20170514T000425_016570_01B7A9_E654
2017/5/20	COPERNICUS/S1_GRD/S1A_IW_GRDH_1SDV_20170520T235553_20170520T235618_016672_01BACE_762B
2017/5/26	COPERNICUS/S1_GRD/S1A_IW_GRDH_1SDV_20170526T000400_20170526T000425_016745_01BD09_E0AB
2017/8/6	COPERNICUS/S1_GRD/S1A_IW_GRDH_1SDV_20170806T000404_20170806T000429_017795_01DD21_4A90
2017/8/12	COPERNICUS/S1_GRD/S1A_IW_GRDH_1SDV_20170812T235557_20170812T235622_017897_01E03B_D81A
2017/8/18	COPERNICUS/S1_GRD/S1A_IW_GRDH_1SDV_20170818T000405_20170818T000430_017970_01E26E_E224
2017/8/24	COPERNICUS/S1_GRD/S1A_IW_GRDH_1SDV_20170824T235558_20170824T235623_018072_01E586_FF38
2017/12/4	COPERNICUS/S1_GRD/S1A_IW_GRDH_1SDV_20171204T000406_20171204T000431_019545_0212ED_685E
2017/12/10	COPERNICUS/S1_GRD/S1A_IW_GRDH_1SDV_20171210T235558_20171210T235623_019647_021620_3550
2017/12/22	COPERNICUS/S1_GRD/S1A_IW_GRDH_1SDV_20171222T235558_20171222T235623_019822_021B82_3A9F
2017/12/28	COPERNICUS/S1_GRD/S1A_IW_GRDH_1SDV_20171228T000405_20171228T000430_019895_021DCF_86B7
2018/1/3	COPERNICUS/S1_GRD/S1A_IW_GRDH_1SDV_20180103T235557_20180103T235622_019997_022104_A50B
2018/1/9	COPERNICUS/S1_GRD/S1A_IW_GRDH_1SDV_20180109T000404_20180109T000429_020070_02234E_D43E
2018/1/15	COPERNICUS/S1_GRD/S1A_IW_GRDH_1SDV_20180115T235557_20180115T235622_020172_022695_004B
2018/1/21	COPERNICUS/S1_GRD/S1A_IW_GRDH_1SDV_20180121T000404_20180121T000429_020245_0228DC_BA64
2018/1/27	COPERNICUS/S1_GRD/S1A_IW_GRDH_1SDV_20180127T235557_20180127T235622_020347_022C20_2467
2018/5/3	COPERNICUS/S1_GRD/S1A_IW_GRDH_1SDV_20180503T235558_20180503T235623_021747_02585F_F520

2018/5/9	COPERNICUS/S1_GRD/S1A_IW_GRDH_1SDV_20180509T000405_20180509T000430_021820_025AB2_4B20
2018/5/15	COPERNICUS/S1_GRD/S1A_IW_GRDH_1SDV_20180515T235559_20180515T235624_021922_025DFC_0FC5
2018/5/21	COPERNICUS/S1_GRD/S1A_IW_GRDH_1SDV_20180521T000406_20180521T000431_021995_026046_A757
2018/5/27	COPERNICUS/S1_GRD/S1A_IW_GRDH_1SDV_20180527T235559_20180527T235624_022097_02638A_D4C3
2018/8/1	COPERNICUS/S1_GRD/S1A_IW_GRDH_1SDV_20180801T000410_20180801T000435_023045_028071_4F28
2018/8/7	COPERNICUS/S1_GRD/S1A_IW_GRDH_1SDV_20180807T235604_20180807T235629_023147_0283AC_6EDB
2018/8/19	COPERNICUS/S1_GRD/S1A_IW_GRDH_1SDV_20180819T235604_20180819T235629_023322_028959_14CB
2018/8/25	COPERNICUS/S1_GRD/S1A_IW_GRDH_1SDV_20180825T000412_20180825T000437_023395_028BA9_E7F2
2018/8/31	COPERNICUS/S1_GRD/S1A_IW_GRDH_1SDV_20180831T235605_20180831T235630_023497_028EE7_2EDD
2018/12/11	COPERNICUS/S1_GRD/S1A_IW_GRDH_1SDV_20181211T000412_20181211T000437_024970_02C09B_B002
2018/12/17	COPERNICUS/S1_GRD/S1A_IW_GRDH_1SDV_20181217T235605_20181217T235630_025072_02C446_3475
2018/12/23	COPERNICUS/S1_GRD/S1A_IW_GRDH_1SDV_20181223T000412_20181223T000437_025145_02C6E9_3750
2018/12/29	COPERNICUS/S1_GRD/S1A_IW_GRDH_1SDV_20181229T235604_20181229T235629_025247_02CAA0_1CDB
2019/1/4	COPERNICUS/S1_GRD/S1A_IW_GRDH_1SDV_20190104T000411_20190104T000436_025320_02CD37_FBC3
2019/1/10	COPERNICUS/S1_GRD/S1A_IW_GRDH_1SDV_20190110T235604_20190110T235629_025422_02D0E9_7826
2019/1/16	COPERNICUS/S1_GRD/S1A_IW_GRDH_1SDV_20190116T000411_20190116T000436_025495_02D389_E00B
2019/1/22	COPERNICUS/S1_GRD/S1A_IW_GRDH_1SDV_20190122T235603_20190122T235628_025597_02D74F_CFEC
2019/1/28	COPERNICUS/S1_GRD/S1A_IW_GRDH_1SDV_20190128T000411_20190128T000436_025670_02D9F3_C032
2019/5/4	COPERNICUS/S1_GRD/S1A_IW_GRDH_1SDV_20190504T000422_20190504T000447_027070_030CB5_863C
2019/5/10	COPERNICUS/S1_GRD/S1A_IW_GRDH_1SDV_20190510T235604_20190510T235629_027172_031023_5388
2019/5/22	COPERNICUS/S1_GRD/S1A_IW_GRDH_1SDV_20190522T235605_20190522T235630_027347_03159F_6103
2019/8/2	COPERNICUS/S1_GRD/S1A_IW_GRDH_1SDV_20190802T235609_20190802T235634_028397_03357D_AEEC
2019/8/8	COPERNICUS/S1_GRD/S1A_IW_GRDH_1SDV_20190808T000417_20190808T000442_028470_0337B5_E35A
2019/8/20	COPERNICUS/S1_GRD/S1A_IW_GRDH_1SDV_20190820T000418_20190820T000443_028645_033DC3_47D1
2019/8/26	COPERNICUS/S1_GRD/S1A_IW_GRDH_1SDV_20190826T235611_20190826T235636_028747_03414E_B972
2019/12/6	COPERNICUS/S1_GRD/S1A_IW_GRDH_1SDV_20191206T000419_20191206T000444_030220_037451_0F77

2019/12/12	COPERNICUS/S1_GRD/S1A_IW_GRDH_1SDV_20191212T235611_20191212T235636_030322_0377DC_1A9C
2019/12/18	COPERNICUS/S1_GRD/S1A_IW_GRDH_1SDV_20191218T000418_20191218T000443_030395_037A62_0627
2019/12/24	COPERNICUS/S1_GRD/S1A_IW_GRDH_1SDV_20191224T235611_20191224T235636_030497_037DE8_E845
2019/12/30	COPERNICUS/S1_GRD/S1A_IW_GRDH_1SDV_20191230T000418_20191230T000443_030570_038063_8FEE
2019/5/4	COPERNICUS/S1_GRD/S1B_IW_GRDH_1SDV_20190504T235513_20190504T235542_016101_01E49E_F422
	COPERNICUS/S1_GRD/S1B_IW_GRDH_1SDV_20190504T235542_20190504T235616_016101_01E49E_4F22
2019/12/12	COPERNICUS/S1_GRD/S1B_IW_GRDH_1SDV_20191212T000354_20191212T000419_019324_0247DB_E233
2019/12/24	COPERNICUS/S1_GRD/S1B_IW_GRDH_1SDV_20191224T000354_20191224T000419_019499_024D6A_48F8
