# Supplementary material for: Quantifying spatio-temporal variation in aquaculture production areas in Satkhira, Bangladesh using geospatial and social survey
Source: PLoS One. 2022 Dec 15;17(12):e0278042. doi: 10.1371/journal.pone.0278042 (PMC9754591; doi:10.1371/journal.pone.0278042)
Supplement: S2 Table — (PDF) [file pone.0278042.s002.pdf]

S2 Table

Accuracy assessment of the Sentinel-1 (SAR) Classification

Table S2_1: Accuracy assessment of the Sentinel-1 (SAR) Classification (2017-01,05,08,12).

Year/Months	2017-01		2017-05		2017-08		2017-12	
Classes/Accuracy	User Accuracy	Producer accuracy	User Accuracy	Producer accuracy	User Accuracy	Producer accuracy	User Accuracy	Producer accuracy
Aquaculture pond	93.33%	80%	90.32%	75.68%	93.1%	84.38%	73.33%	88%
Paddy-field	80%	96%	79.31%	92%	86.67%	89.66%	90%	72.97%
Paddy-field with fish	90%	90%	80%	88.89%	80.95%	89.47%	83.33%	100%
Overall Accuracy	87.5%		83.75%		87.5%		81.94%	

Table S2_2: Accuracy assessment of the Sentinel-1 (SAR) Classification (2018-01,05,08,12).

Year/Months	2018-01		2018-05		2018-08		2018-12	
Classes/Accuracy	User Accuracy	Producer accuracy	User Accuracy	Producer accuracy	User Accuracy	Producer accuracy	User Accuracy	Producer accuracy
Aquaculture pond	93.55%	82.86%	93.33%	82.35%	90%	84.38%	80%	82.76%
Paddy-field	93.33%	84.85%	83.33%	86.21%	86.67%	74.29%	83.33%	75.76%
Paddy-field with fish	68.18%	100%	60%	75%	65%	100%	70%	87.5%
Overall Accuracy	86.75%		82.67%		82.5%		80%	

Table A3: Accuracy assessment of the Sentinel-1 (SAR) Classification (2019-01,05,08,12).

Year/Months	2019-01		2019-05		2019-08		2019-12	
Classes/Accuracy	User Accuracy	Producer accuracy	User Accuracy	Producer accuracy	User Accuracy	Producer accuracy	User Accuracy	Producer accuracy
Aquaculture pond	83.33%	92.59%	81.25%	89.66%	86.67%	96.3%	75.86%	88%

Paddy-field	100%	70%	96.67%	85.29%	100%	83.33%	79.31%	88.46%
Paddy-field with fish	73.33%	91.67%	84.62%	91.67%	75%	100%	95.45%	72.41%
Overall Accuracy	84.75%		88%		90.28%		82.5%	
