# Supplementary material for: Quantifying spatio-temporal variation in aquaculture production areas in Satkhira, Bangladesh using geospatial and social survey
Source: PLoS One. 2022 Dec 15;17(12):e0278042. doi: 10.1371/journal.pone.0278042 (PMC9754591; doi:10.1371/journal.pone.0278042)
Supplement: S1 File — (PDF) [file pone.0278042.s003.pdf]

Supplementary file S1

GEE code to generate MNDWI data and maps

```
Map.addLayer(Sathkira,{},'Sathkira',false);
function maskS2clouds(image) {
  var qa = image.select('QA60');

  // Bits 10 and 11 are clouds and cirrus, respectively.
  var cloudBitMask = 1 << 10;
  var cirrusBitMask = 1 << 11;
  // Both flags should be set to zero, indicating clear conditions.
  var mask = qa.bitwiseAnd(cloudBitMask).eq(0)
    .and(qa.bitwiseAnd(cirrusBitMask).eq(0));

  return image.updateMask(mask);
}
var image = ee.ImageCollection('COPERNICUS/S2')
                .filterDate('2017-01-01', '2017-01-31')
                .filterBounds(Sathkira)
                .map(maskS2clouds)
                .median()
                ;
    //var thrsValue = 0.4;
print(image);
var visParams = {
  bands: ['B4', 'B3', 'B2'],
  min: 0,
  max: 3000,
  gamma: 1.4,
};

image = image.clip(Sathkira);
```

```
//Map.addLayer(image, visParams);
var imageTOA = image.divide(10000);
//Add true-clour composite to map
//Map.addLayer(image, {bands: ['B4', 'B3', 'B2'],min:0, max: 3000}, 'True colour image');
Map.addLayer(imageTOA, {bands: ['B4', 'B3', 'B2'],min:0, max: 0.3000}, 'True colour image');
var mndwi = image.expression(
   'float(GRN - SWIR) / float(GRN + SWIR)', {
     'SWIR': image.select('B11'),
     'GRN': image.select('B3'),
   });
// var ndwi = image.expression(
//     'float(GRN - NIR) / float(GRN + NIR)', {
//       'NIR': image.select('B8'),
//       'GRN': image.select('B3'),
//     });
// var mndwi = ndwi;
//////////OTSU///////////////////////////
var histogram = mndwi.reduceRegion({
  reducer: ee.Reducer.histogram(255, 2)
      .combine('mean', null, true)
      .combine('variance', null, true),
  geometry: Sathkira,
  scale: 30,
  bestEffort: true
});
print(histogram,'histogram');
// Chart the histogram
print(Chart.image.histogram(mndwi, Sathkira, 30));
var otsu = function(histogram) {
  var counts = ee.Array(ee.Dictionary(histogram).get('histogram'));
  var means = ee.Array(ee.Dictionary(histogram).get('bucketMeans'));
  var size = means.length().get([0]);
```

```
  var total = counts.reduce(ee.Reducer.sum(), [0]).get([0]);
  var sum = means.multiply(counts).reduce(ee.Reducer.sum(), [0]).get([0]);
  var mean = sum.divide(total);
   var indices = ee.List.sequence(1, size);
   // Compute between sum of squares, where each mean partitions the data.
  var bss = indices.map(function(i) {
   var aCounts = counts.slice(0, 0, i);
   var aCount = aCounts.reduce(ee.Reducer.sum(), [0]).get([0]);
   var aMeans = means.slice(0, 0, i);
   var aMean = aMeans.multiply(aCounts)
       .reduce(ee.Reducer.sum(), [0]).get([0])
       .divide(aCount);
   var bCount = total.subtract(aCount);
   var bMean = sum.subtract(aCount.multiply(aMean)).divide(bCount);
   return aCount.multiply(aMean.subtract(mean).pow(2)).add(
          bCount.multiply(bMean.subtract(mean).pow(2)));
  });

  print(ui.Chart.array.values(ee.Array(bss), 0, means));

  // Return the mean value corresponding to the maximum BSS.
  return means.sort(bss)
  .get([0])
  ;
};
var threshold = otsu(histogram.get('B3_histogram'));
print('threshold', threshold);

//var WaterBody1 = mndwi.gt(threshold); // You can change value here
//////////////////////////////////////////////////////////////////

// var ndwimask = mndwi.gt(threshold);
```

```
// mndwi = mndwi.updateMask(ndwimask);
mndwi = mndwi.gt(threshold).selfMask();


Map.addLayer(mndwi, {min:-1, max: 1, palette: ['brown', 'red',
'orange','yellow','green','cyan','blue','indigo','white']}, 'MNDWI');
Export.image.toDrive(
  {
  image:mndwi.clip(Sathkira),
  description: 'MNDWI_',
  scale: 10,
  region: Sathkira,
   crs: 'EPSG:4326',
   folder: 'Satkira_water'
//  maxPixels:34089663741
  }  )
// // Pre-define some customization options.
// var options = {
//   title: 'Histogram chart',
//   fontSize: 15,
//   hAxis: {title: 'MNDWI value'},
//   vAxis: {title: 'count of Pixels'},
//   series: {
//     0: {color: 'Blue'},
// }};
// // Make the histogram, set the options.
// var histogram = ui.Chart.image.histogram(mndwi, Sathkira, 30)
//     .setSeriesNames([ 'MNDWI'])
//     .setOptions(options);
// // Display the histogram.
// print(histogram);
var samples = ee.FeatureCollection(Sathkira);
// Create the scatter chart
var Chart1 = ui.Chart.image.regions(
```

```
    mndwi, samples, ee.Reducer.mean(), 10, 'label')
        .setChartType('ScatterChart');
print(Chart1);

/////////Area calculator///////////////
var count = mndwi;
var total = count.multiply(ee.Image.pixelArea());
var area = total.reduceRegion({
    reducer:ee.Reducer.sum(),
    geometry:Sathkira,
    scale:30,
    maxPixels: 1e9,
    bestEffort:true
});
var area_pxa = ee.Number(area);
//.divide(1e6)
//print ('Area in (m²):', area_pxa);
//////////////////////////////////
// km square
//var img = ee.Image.pixelArea().divide(1000000);

var reducer = mndwi.reduceRegion({
  reducer: ee.Reducer.count(),
  geometry: Sathkira,
//  crs: 'EPSG:32645', // WGS Zone N 45
  scale: 30,
//  maxPixels: 1E13
});
print(reducer,'pixel count');
// gives an area of 147134.49 km2
//var area = cnt.multiply(30).multiply(30).divide(1000000);
//print('Area of zone using pixel count method: ', area.getInfo() + ' km2');
```
