# Supplementary material for: Quantifying spatio-temporal variation in aquaculture production areas in Satkhira, Bangladesh using geospatial and social survey
Source: PLoS One. 2022 Dec 15;17(12):e0278042. doi: 10.1371/journal.pone.0278042 (PMC9754591; doi:10.1371/journal.pone.0278042)
Supplement: S2 File — (PDF) [file pone.0278042.s004.pdf]

Supplementary file S2

QUESTIONNAIRE SURVEY FOR AQUACULTURE AND SHRIMP FARMING

SATKHIRA, BANGLADESH

Dear Sir/Madam,

My name is Nujaira Hafeza. I am Master's student of Graduate School of Environment Science, Hokkaido University, Japan. I am doing a research about Satellite Remote Sensing. My research topic is to monitor water-logging area specially aquaculture and Shrimp farming, Satkhira, Bangladesh. Could you please give me about 15 minutes to fill out this questionnaire? All information in this questionnaire will be considered as confidential and will be used research purpose only.

Date of interview: .... Affiliation…………………………………

Village, community: .... Interviewee: ....

1. What you do in your field? Rice cultivate/ aquaculture / rice and aquaculture(both)/ other

   How often/how many times you cultivate rice in a year?

Aman-June-Jan Aus.- March -Aug Boro-Jan-Apr

If Aquaculture what kind of fish you are cultivate? Shrimp farming/ others

If both when and how?

2. How long you do Aquaculture? Why? —
3. Motivation for doing aquaculture? Friends/ Family/ others
4. Why you select/ interested to do Aquaculture (shrimp farming)?
5. Do you thing aquaculture (water-logging) is: good /bad?
   Why?……………..
6. In which water you do shrimp farming? Fresh water/ salty water
7. What time is suitable for shrimp farming? For how long?
8. Do you think shrimp farming is suitable for this area? Yes/No
   Why?.....................
9. Why shrimp are cultivated in this field?
10. How many days you are engaged for doing shrimp farming?
11. Previous profession/earning sources?
12. Who else is involved for shrimp farming from your family?
13. Are you satisfied with your work? Yes/No
    (if No why?)…………..
14. Do you think for doing shrimp farming poverty is increasing/decreasing?
15. What is the challenge of aquaculture/ shrimp farming?
16. What kind of problem are increasing doing salt water shrimp farming?

17. What do you think, which problem is the most for environment?
    a. Water pollution
    b. Land subsidence
    c. Coastal erosion/flooding
    d. Solid wastes
    e. Other: ....
18. What is the solution/how can we solve this problem?
19. For salinity is there any problem to cultivate other crops in future? What do you think?

…………………………………….

20. What you are thinking shrimp farming is more profitable then agriculture?
21. For water-logging/ aquaculture the crops are affected?
22. In the last 12 months, what benefits has your family gained from the Shrimp farming?
23. Please list down your recommendations to improve:

No	Aquaculture	Shrimp Farming
1		

THANK YOU FOR YOUR KIND COLLABORATION!

My purpose

How many people are involved for doing aquaculture specially shrimp farming (salt water)?

What kind of environmental problem are increasing for doing shrimp farming?
