# Supplementary material for: Quantifying spatio-temporal variation in aquaculture production areas in Satkhira, Bangladesh using geospatial and social survey
Source: PLoS One. 2022 Dec 15;17(12):e0278042. doi: 10.1371/journal.pone.0278042 (PMC9754591; doi:10.1371/journal.pone.0278042)

S1 Figure

Pictures collected during the field visit shows diverse types of aquaculture in the study area.

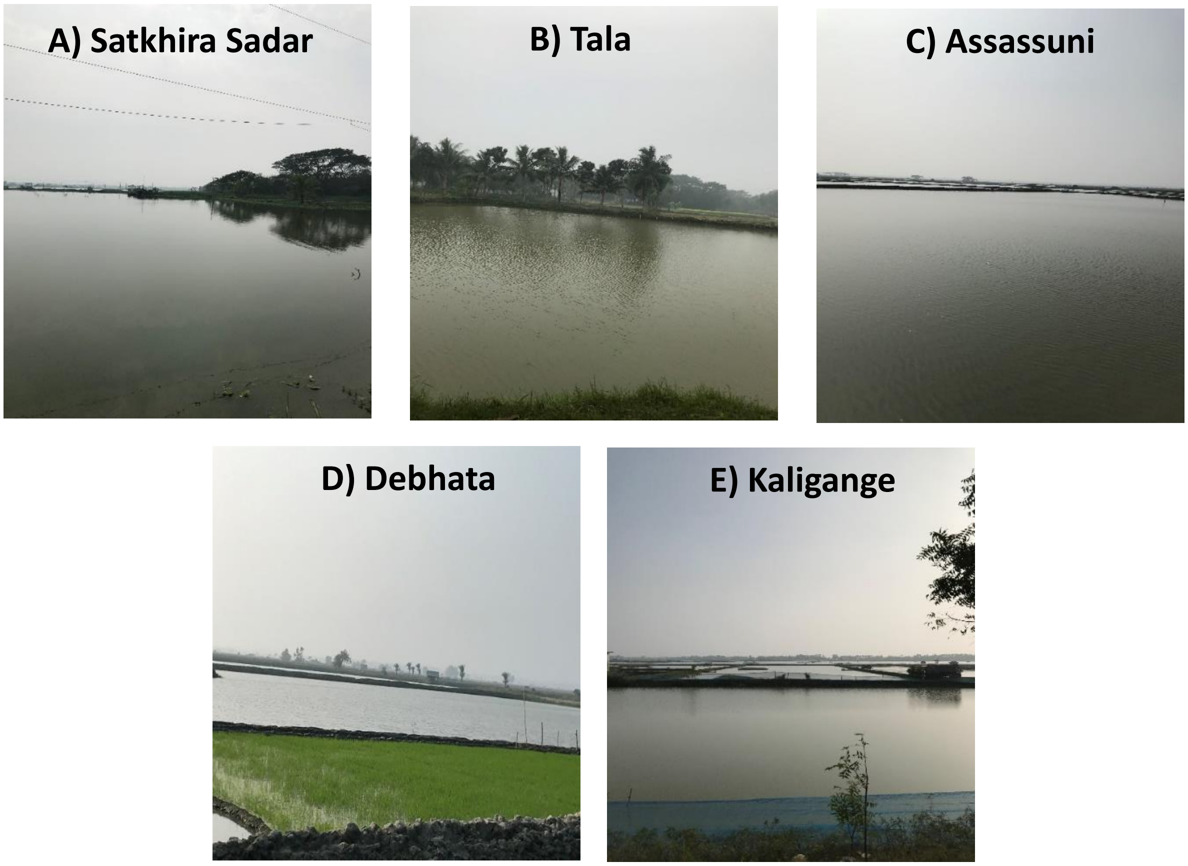

Supplement: S1 Fig — (PDF) [file pone.0278042.s005.pdf]
