# Supplementary material for: Quantifying spatio-temporal variation in aquaculture production areas in Satkhira, Bangladesh using geospatial and social survey
Source: PLoS One. 2022 Dec 15;17(12):e0278042. doi: 10.1371/journal.pone.0278042 (PMC9754591; doi:10.1371/journal.pone.0278042)
Supplement: Attachment — Submitted filename: reply to reviewers comments_nujaira.docx [file pone.0278042.s006.docx]

 Dear Editor,

We very much appreciate all the reviewers and editor for encouraging, critical and constructive comments on this manuscript. The comments and suggestions have been extensive and useful to improve the manuscript. We strongly believe that these comments and suggestions have increased the scientific value of the revised manuscript by many folds. We have taken them fully into account in revision. We are submitting the revised version of the manuscript with the suggestion incorporated in the manuscript. The manuscript has been revised as per the comments given by the reviewer, and our responses to all the comments are as follows:

**Reviewer #1:** The paper is good, but it needs some enhancements:

**Reply : Thank you so much for your useful comments and suggestion. We greatly appreciate them for making our manuscript better.**

- Figures quality not good. Improve them.

**Reply: Thank you very much for your suggestion. We have revised the figures and improved the quality of manuscript.**

- English has typos.

**Reply: We have revised the manuscript and revised English thoroughly.**

- More details about the employed methods can be added.

**Reply: Thank you very much for your suggestion. We have revised the methodology and added more details in the revised version of the manuscript. (Line: 158-340)**

- Elaborate your motivation, contribution in the introduction section.

**Reply: Thank you very much for your suggestion. We have revised the motivation and contribution in the introduction part. (Line: 103-124 )**

-The conclusion must be improved. The authors should focus on their unique work and contributions at first, and they should support their conclusion by numerical results. Then, the limitations of this paper should be discussed. Accordingly, the future work of this paper can be drawn;

**Reply: Thank you very much for your kind suggestion. We have thoroughly revised the conclusion and included limitations and future scope of this study in the revised version of the discussion (Line: 617-627 )**

**Reviewer #2:** This study doesn't include a proposal for a paper for the journal Plos One.
The theme is interesting, but it has little data (temporal and spatial) and incomplete statistical evaluation to be published in a scientific journal like a Plos One.
I recommend submitting the work to a symposium, congress or similar.

**Reply: Thank you very much for your suggestion. We have clearly mentioned motivation and contribution of this manuscript in the introduction part of the manuscript. This study is important for data scarce countries like Bangladesh. This study will provide innovative approach to monitor aquaculture areas using freely available geospatial data to improve aquaculture management. As aquaculture contribute towards GDP it is essential to manage them sustainable. Hope you will find them justified.**

**Reviewer #3:** The title of the manuscript is interesting but there are some major concerns need to be addressed are as follow:

**Reply: Thank you so much for your useful comments and suggestion. We greatly appreciate them for making our manuscript better.**

1. The novelty of the manuscript is questionable. Authors need to highlight “what is the exact novelty of this manuscript ?” and in a better way, it needs to be presented in enumerate points.

**Reply : Thank you so much for your useful suggestion. We have revised the motivation and contribution in the introduction part to reflect the novelty of this manuscript. (Line: 103-124)**

2. There is no description regarding the splitting of the dataset and how they used to evaluate the experiment ? because it is important for another researcher to replicate that experiment.

**Reply: Thank you very much for your suggestion. We have revised the methodology and added more details in the revised version of the manuscript. It will help other researches to replicate the methodology in other study areas. (Line: 158-340 )**

3. There are some statistical parameters used in this study for the evaluation but there is a description as well as numerical presentation required.

**Reply: Thank you very much for your suggestion. We have revised the methodology and added numerical formulas that has been used in this study in the revised version of the manuscript.**

4. What is the experimental testbed used to achieve that output ? It should be hardware and software both required.

**Reply: Thank you very much for your suggestion. We have included information about softwares used in this study that can other researchers to follow the methodology.**

5. There are introductory lines required between the section and subsection to make flow for the readers.

**Reply: Thank you very much for your suggestion. We have thoroughly revised the introduction part of the manuscript to make it more coherent.**
