# Supplementary material for: Quantifying spatio-temporal variation in aquaculture production areas in Satkhira, Bangladesh using geospatial and social survey
Source: PLoS One. 2022 Dec 15;17(12):e0278042. doi: 10.1371/journal.pone.0278042 (PMC9754591; doi:10.1371/journal.pone.0278042)
Supplement: Attachment — Submitted filename: R2_reply to reviewers comments.docx [file pone.0278042.s007.docx]

**Dear Editor and reviewers,**

**We very much appreciate all the reviewers and editor for encouraging, critical and constructive comments on this manuscript. The comments and suggestions have been extensive and useful to improve the manuscript. We strongly believe that these comments and suggestions have increased the scientific value of the revised manuscript by many folds. We have taken them fully into account in revision. We are submitting the revised version of the manuscript with the suggestion incorporated in the manuscript. The manuscript has been revised as per the comments given by the reviewer, and our responses to all the comments are as follows:**

**Reviewer# 1**

| No. | Comments | Responses |
| --- | --- | --- |
|  |  |  |
| 1 | I recommend inserting the information obtained, in addition to that mentioned in the article, about secondary data and the questionnaire applied in December 2019. | We once again thank you very much for your time and constructive feedback. Following your suggestion, we now incorporate the questionnaire and survey response transcript as supplementary material with this paper. We also mentioned it in the section 4.3. The questionnaire survey form and results based on questionnaire survey is provided in supplementary file S1 and supplementary file S2, respectively. |
| 2 | I recommend inserting radiometric and atmospheric corrections into Sentinel 2 data. | We added the following:  See line 174-187  “We obtained the pre-processed surface reflectance Sentinel-2 L2A data from GEE through scihub. They were initially computed by running Sen2Cor processor, consists in scene classification and atmospheric correction applied to Level-1C orthoimage product. Atmospheric correction in Sen2Cor is performed using a set of look-up tables generated via libRadtran. The aerosol type and visibility or optical thickness of the atmosphere is derived using the Dense Dark Vegetation (DDV) algorithm. Clouds if any present in the scene are removed by using COPERNICUS/S2_CLOUD_PROBABILITY algorithm. A total of 380 Sentinel-2 scenes available between 2017 January and 2019 December were processed in GEE for this study (See Supplementary file S3)”. |
| 3 | I recommend entering how many scenes from sentinel 1 and 2 were used between January 2017 to December 2019 | Thank you. We added this information in revised manuscript.  See Line 165-170 A total of 56 Sentinel-1 scenes available between 2017 January and 2019 December were used in this study (See Supplementary file S3). |
| 4 | I also recommend adding the temporal range in 3.1.2, in order to facilitate the reading | Added:  See Line 178-180: A total of 380 Sentinel-2 scenes available between 2017 January and 2019 December were processed in GEE for NDWI and MNDWI extraction (See supplementary file F3). |
| 5 | I recommend detailing in 3.3 the pre-processing procedures performed on sentinel data 1 and 2. | We revised the section 3.3 for clarity. Processing codes for NDWI and MNDWI are now provided in the data availability section.  See the code for MNDWI extraction from S2 L2A in GEE: Supplementary file S4  Pre-processing procedure for S1 and S2 are now presented in section 3.1 |
| 6 | I recommend entering the results of non-parametric statistical analysis in 3.3.2 (referring to Figure 4), and 4.2.1 (referring to Figure 10). Do not forget to include the analyzed methodology of these analyses. | Thank you very much. We added the details of non-parametric tests – referring to fig 4 in section 3.3.2. See Line 262-275”  *For each shape metric we used, the non-parametric Mann Whitney U test (also known as Wilcoxon rank sum test) was conducted to see any significant difference in the metric values between the 5 sites. The analysis was done in R language v. 4.1.3 with the ‘wilcox_test()’ function in the package ‘rstatix’. The test was conducted for each combination of sites, with null hypothesis that there is no shift in the distribution of site 1 and 2, and alternative hypothesis is that group 1 is shifted to the left of group 2. We noticed that for the area metrics, Satkhira Sadar had significantly larger ponds than Assassuni (p < 0.001), Kaligange (p = 0.009) and Debhata (p = 0.026). However, Satkhira Sadar’s pond perimeter was only significantly larger than those of Assassuni (p = 0.039). For the compactness metrics, Debhata had significantly higher values than Assassuni (p < 0.001) and Kaligange (p <0.001), and similarly Satkhira Sadar had significantly higher values than Assassuni (p = 0.006) and Kaligange (p = 0.008). For the P2A metric, Kaligange was higher than Debhata (p < 0.001) and Satkhira Sadar (p = 0.08), and Assassuni was also higher than Debhata (p < 0.001) and Satkhira Sadar (p = 0.06). All reported p-values are adjusted for multiple comparison using the Holm method”.*  Because of limited datapoints, no non-parametric test was conducted for Fig.10. |
| 7 | # I recommend not using the kappa index. Since 2011, the Remote Sensing community has avoided using this index. See the article: [https://doi.org/10.108 0/01431161.2011.552923](https://doi.org/10.108%200/01431161.2011.552923). | We agree with the reviewer here. As reported in Stehmen and Foody, 2019 (Remote Sensing of Environment).. “Liu et al. (2007) showed that kappa was highly correlated with overall accuracy, which is evident from Eq. (24), so reporting both measures is redundant”. ………. ”………….. it may cause no serious harm if you have it and pay little attention to it, but it does not fulfil a necessary function.”. <https://doi.org/10.1016/j.rse.2019.05.018>  Thus, the accuracy of our results can be noted from the overall accuracy and kappa values were eliminated. |
| 8 | I recommend inserting the NDWI and MNDWI maps to winter and pre-monsoon seasons (Figure 7) and the same season for Figure 8. | Thank you very much for your kind suggestion. As we have generated NDWI and MNDWI data using Sentinel-2 data in google earth engine. As Bangladesh is a tropical country and Sentinel-2 was full of clouds during the pre-monsoon and monsoon months therefore, we didn’t use in this analysis. However, we checked the trend of the aquaculture area using Sentinel-1 (SAR) data to overcome the limitations of clouds. |

**Reviewer# 2**

| No | Comments | Responses |
| --- | --- | --- |
| 1 | Authors need to mention in the introduction what techniques are used in this research as well as the novelty of the manuscript mentioned pointwise. | Thanks for the time and constructive suggestions. Following your comment, we revised the introduction section for clarity.  See Line 101-120  *Although Bangladesh plays an important role in the aquaculture sector and contributes significantly to the national GDP, the lack of up-to-date, explicit and continuous spatial knowledge about aquaculture imposes a great hurdle in its sustainable management. Here, we employed multi-temporal Sentinel-1 and Sentinel-2 images from 2017-2019 to track the changes in aquaculture productivity in the Satkhira district, Bangladesh. This study focuses on providing a holistic picture of factors responsible for the trend in aquaculture, its related opportunities and challenges for people in the Satkhira district. The main objectives of this study are: (a) Monitor the spatio-temporal extent of aquaculture ponds from 2017 to 2019 in Satkhira district using an integrated geospatial and field approach; and (b) provide detailed information on socio-economic perspectives on aquaculture in Satkhira, Bangladesh, to enable a more sustainable and profitable management. While the first objective can be achieved by quantitative remote sensing approach, the second objective is qualitative and used key informant interviews with the relevant stakeholders in the region. T*  *This study will be useful to identify not only the spatio-temporal variation but as well problems associated with the aquaculture industry. It also proposes a possible management solution that can be beneficial for common farmers and other stakeholders, such as government and NGOs. In addition, this research will play an important role for the government in achieving the SDG goals. In particular, mapping and quantification of existing aquaculture areas can contribute to food security (SDG 2); clean water and sanitation (SDG 6.0), economic growth and better livelihoods (SDG 8); and sustainable consumption (SDG 12), to name a few.* |
| 2 | Comment 3 & 4, where authors highlight the statistical parameters formula and software details respectively? | Thank you very much. We added the details of non-parametric tests – referring to fig 4 in section 3.3.2. See Line 259-272”  *For each shape metric we used, the non-parametric Mann Whitney U test (also known as Wilcoxon rank sum test) was conducted to see any significant difference in the metric values between the 5 sites. The analysis was done in R language v. 4.1.3 with the ‘wilcox_test()’ function in the package ‘rstatix’. The test was conducted for each combination of sites, with null hypothesis that there is no shift in the distribution of site 1 and 2, and alternative hypothesis is that group 1 is shifted to the left of group 2. We noticed that for the area metrics, Satkhira Sadar had significantly larger ponds than Assassuni (p < 0.001), Kaligange (p = 0.009) and Debhata (p = 0.026). However, Satkhira Sadar’s pond perimeter was only significantly larger than those of Assassuni (p = 0.039). For the compactness metrics, Debhata had significantly higher values than Assassuni (p < 0.001) and Kaligange (p <0.001), and similarly Satkhira Sadar had significantly higher values than Assassuni (p = 0.006) and Kaligange (p = 0.008). For the P2A metric, Kaligange was higher than Debhata (p < 0.001) and Satkhira Sadar (p = 0.08), and Assassuni was also higher than Debhata (p < 0.001) and Satkhira Sadar (p = 0.06). All reported p-values are adjusted for multiple comparison using the Holm method”.*  We additionally explained in section 3.1 and 3.3 the pre-processing methods carried with the satellite images and software employed. |
